# Five tree species contained antibiotic-producing bacteria within their bark

**DOI:** 10.17912/micropub.biology.001227

**Published:** 2024-08-01

**Authors:** Caymen Hoffman, Kristina Blanke

**Affiliations:** 1 Biology Department, Beloit College, Beloit, Wisconsin, United States; 2 Pakula Biomedical Fellowship

## Abstract

Soil is a common source for identifying antibiotic-producing bacteria; however, other ecosystems in nature may contain novel bacteria capable of producing antibiotics. Bark from seven tree species was collected as a new source to culture bacterial isolates that were screened against nine tester bacteria related to antibiotic resistant pathogens. Five of the seven tree species contained isolates that showed antibiotic production against at least one of the tester bacteria. Bark should be further explored as a possible source for bacteria that produce unknown antibiotics.

**Figure 1. Bacterial colony morphologies from six tree species and select isolates screened against tester bacteria with visible growth inhibition:**
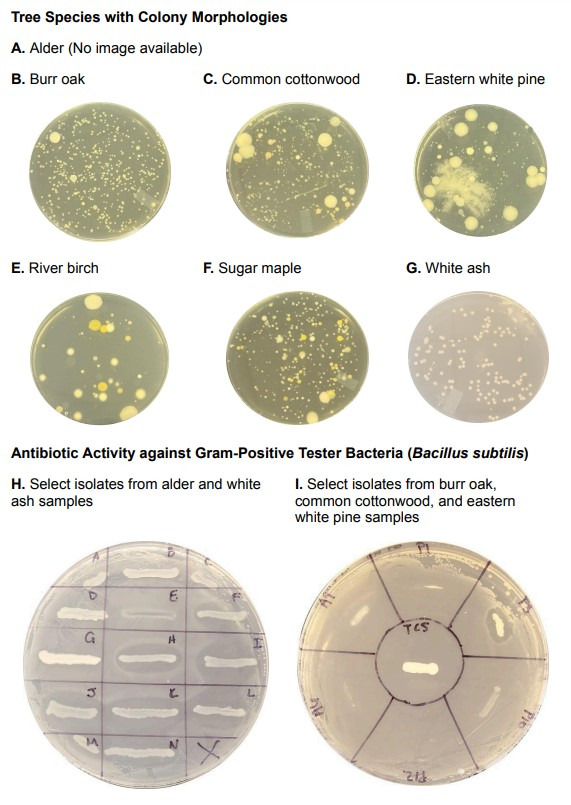
Each tree species is identified and the images show the colony variation across bacteria cultured from the bark (A-G). Tree species were identified with the Pl@ntNet mobile app. **A.** Alder ( *Alnus glutinosa* ) Image was not available. **B.** Burr oak ( *Quercus macrocarpa* ) **C.** Common cottonwood ( *Populus deltoides* ) **D.** Eastern white pine ( *Pinus strobus* ) **E.** River birch ( *Betula nigra* ) **F.** Sugar maple ( *Acer saccharum* ) **G.** White ash ( *Fraxinus americana* ) **H.** Alder and white ash isolates were screened against *Bacillus subtilis* . Isolates F, H, I, J, K, L, M, and N from the white ash bark show zones of inhibited growth for the tester bacteria. **I.** Burr oak, common cottonwood, and eastern white pine isolates were screened against *Bacillus subtilis* and the P3 isolate from the eastern white pine bark shows inhibited growth.

## Description

Soil is an ideal ecosystem to create competition among resident bacteria that need to obtain resources from the environment. Bacteria increase their likelihood to gain resources by creating secondary metabolites, or antibiotics, to inhibit or kill neighboring bacteria. The secondary metabolites could be effective antibiotics to stop the global healthcare crisis of antibiotic resistant pathogens. Soil is the most common source of antibiotic-producing bacteria because it contains diverse amounts of actinomycetes (Sapkota et al., 2020) , the most common group of bacteria that produces antibiotics. Unfortunately, soil environments are not cultivating bacteria that produce new antibiotics at an equivalent pace to the rise of antibiotic resistant pathogens. Therefore, alternative habitats for actinomycetes have been identified in coastal marine environments (Stevens et al., 2007) , moonmilk from caves (Maciejewska et al., 2018) , and in a symbiotic relationship with ants (Poulsen et al., 2005) . These studies emphasize the need to explore new natural sources for antibiotic-producing bacteria.

This study expanded the search to biotic sources and aimed to determine if tree bark contained antibiotic-producing bacteria. Seven tree species were located across Central and Southern Wisconsin, and bark from one tree per species was collected and serial diluted before culturing bacteria. Select bacterial isolates were chosen from each tree species and screened against nine tester bacteria that are close relatives to antibiotic resistant pathogenic bacteria.

Antibiotic-producing bacteria were cultured from five of the seven bark samples associated with the following tree types: alder, burr oak, common cottonwood, eastern white pine, and white ash (Table 1). Certain isolates inhibited the growth of all three categories of tester bacteria, while other isolates were specific to either Gram-negative or Gram-positive bacterial inhibition. Bacterial colonies from all five tree types had similar morphologies ( Figure 1 . A-D, G) with a glossy or smooth surface, various colors (cream, pink, tan, white, yellow), circular form, entire margin, and a few elevation types (convex, flat, raised). The antibiotic-producing bacteria had similar morphological characteristics across bark samples. Both the alder and white ash had 36% of their isolates producing antibiotics ( Figure 1 . H), and the eastern white pine had 33% of its isolates producing antibiotics ( Figure 1 . I). These three tree species had high numbers of antibiotic-producing bacteria, indicating that the barks contain ecosystems where bacteria may need to compete for resources and in turn produce antibiotics. The burr oak had 17% and the common cottonwood had 8% of their isolates producing antibiotics, which may indicate less bacterial competition on these bark samples.

**Table:** 

**Table 1.** Tree species with corresponding colony forming units (CFU) per gram of sample, the total number of isolates tested and the number with antibiotic production from each sample set, and the number of isolates that inhibited growth of Gram-negative, Gram-positive, or acid fast stained tester bacteria. River birch and sugar maple isolates did not produce antibiotics against the tester bacteria.						
**Tree Species**	**CFU/g**	**Total Number of Isolates Tested**	**Number of Isolates with Antibiotic Activity**	**Gram-Negative**	**Gram-Positive**	**Acid Fast**
Alder	5.1 x 10 ^6^	11	4	3	4	2
Burr oak	4.63 x 10 ^5^	12	2	1	2	0
Common cottonwood	4.1 x 10 ^6^	12	1	0	1	1
Eastern white pine	5.3 x 10 ^4^	12	4	2	4	2
White ash	1.48 x 10 ^6^	22	8	0	8	8
River birch	7.2 x 10 ^3^	12	0	∅	∅	∅
Sugar maple	2.65 x 10 ^6^	12	0	∅	∅	∅

Neither the river birch nor sugar maple contained antibiotic-producing bacteria. The river birch sample had the lowest number of colony forming units, but the colonies had similar morphological diversity to the sugar maple colonies ( Figure 1 . E-F) with a range of surfaces (dry, dull, fuzzy, glistening, mucoid, rough, smooth, wrinkled), colors (brown, cream, pink, red, tan, white, yellow), margins (entire, irregular, undulate), and elevation (convex, flat, pulvinate, raised, umbonate). These two bark samples contained the most diverse colony morphologies yet the bacteria did not produce antibiotics, which may indicate there is less competition on these bark since the micro-ecosystems included more bacterial species.

The low number of initial isolates screened from each bark sample and sampling from a single individual of each tree species limits the inference that bacteria on river birch and sugar maple trees do not produce antibiotics. Twelve colonies were chosen across the serial dilutions for each tree species based on visible differences among colonies; then, isolates were screened against all nine tester bacteria. There were numerous colonies and this initial study did not screen all possible isolates. Therefore, river birch and sugar maple bark may contain antibiotic-producing bacteria.

These results show that bark is a viable option for discovering antibiotic-producing bacteria. More tree species should be surveyed for bacterial profiles and the production of antibiotic metabolites. The current list of tree species should be further studied for a comprehensive understanding of their bark microbiome and identification of new bacterial species with beneficial secondary metabolites. Soil and other natural habitats are valuable resources that should be utilized to better understand how organisms interact, specifically in the realm of bacterial competition.

In conclusion, bark samples from five different individual tree species contained isolates with antibiotic activity against relatives of antibiotic resistant pathogens. Therefore, bark should be more thoroughly explored for novel antibiotic-producing bacteria.

## Methods

Sample Collection and Bark Preparation

Tree species were identified with the Pl@ntNet mobile app and bark samples from the exterior of each tree were collected. Only one tree was sampled per tree species. Bark samples were ground and 1 gram of the sample was used to create serial dilutions (Hernandez et al., 2018) . Bacteria were cultured on complex media (20% tryptic soy agar, lysogeny broth agar, or nutrient agar) and incubated around 27°C for 24-48 hours.

Colony Counts and Morphologies

Colony forming units were calculated for each tree species sample from serial dilutions that contained 30-100 colonies. Additional testing was conducted on colonies selected for differences in size, surface, color, form, margin, and elevation. Any tree species with fewer than 12 morphologically distinct colonies included colonies that were visibly similar. Twelve colonies were selected for each tree species; however, not all isolates grew during the screens and led to fewer total isolates screened. The white ash sample started with 24 selected colonies since the majority of the bacteria were visibly similar.

Antibiotic Activity

The sample set of isolates chosen from each tree species was screened against nine tester bacteria related to pathogens. Five organisms are Gram-negative: *Acinetobacter baylyi* (ATCC # 33305), *Enterobacter aerogenes* (ATCC # 51697), *Erwinia carotovora* (ATCC # 25270), *Escherichia coli* (ATCC # 25922), *Pseudomonas putida* (Handelsman Lab strain, original ATCC # 47054). Three organisms are Gram-positive: *Bacillus subtilis* (Handelsman Lab strain, original ATCC # 6051), *Enterococcus raffinosus* (ATCC # 49464), *Staphylococcus epidermidis* (ATCC # 14990). One organism requires acid fast staining: *Mycobacterium smegmatis* (ATCC # 700084). All screens were completed on complex media (20% tryptic soy agar, lysogeny broth agar, or nutrient agar) and incubated around 25°C for about 48 hours. Zones of inhibition around the isolates were verified by at least two researchers to determine if there was or was not a zone of inhibition visible against a lighted background.
